# Supplementary material for: Protocol for a feasibility study incorporating a randomised pilot trial with an embedded process evaluation and feasibility economic analysis of ThinkCancer!: a primary care intervention to expedite cancer diagnosis in Wales
Source: Pilot Feasibility Stud. 2021 Apr 21;7:100. doi: 10.1186/s40814-021-00834-y (PMC8059131; doi:10.1186/s40814-021-00834-y)
Supplement: Supplementary file 1 — Additional file 1:. WHO Trial Registration Data Set. [file 40814_2021_834_MOESM1_ESM.pdf]

Trial Registration – data set (from the WHO Trial Registration Set)

**Data Category**	**Information**
Primary registry and trial identifying number	Intended: ClinicalTrials.gov - *trial identifying number pending*
Date of registration in primary registry	N/A - pending
Secondary identifying numbers	IRAS 256824
Source(s) of monetary or material support	Cancer Research Wales
Primary sponsor	Bangor University
Contact for public queries	SD (stefanie.disbeschl@bangor.ac.uk)
Contact for scientific queries	N/A
Public title	Protocol for a Feasibility Study of ThinkCancer! – A primary care intervention to expedite cancer diagnosis in Wales
Scientific title	Protocol for a Feasibility study incorporating a Randomised Pilot Trial with an Embedded Process Evaluation and Feasibility Economic Analysis of ThinkCancer!: A primary care intervention to expedite cancer diagnosis in Wales
Countries of recruitment	Wales
Health condition(s) or problem(s) studied	Primary care approaches to cancer referral and safety netting
Intervention(s)	ThinkCancer! intervention (educational behaviour change workshop)
	Control: usual care
Key inclusion and exclusion criteria	All general practices in Wales are invited to participate, with the general practice team being the participant
	No exclusion criteria
Study type	Interventional, phase II
	Allocation: randomized intervention model, ratio 2:1 (intervention vs. control).
	Primary purpose: assess feasibility of delivering the intervention in Wales
Date of first enrolment	March 2020
Target sample size	23-30
Recruitment status	Recruiting
Primary outcome(s)	Feasibility, two week wait referral rate and primary care interval
Key secondary outcomes	Detection rate and conversion rate
