# Supplementary figures and images for: Protocol for a feasibility study incorporating a randomised pilot trial with an embedded process evaluation and feasibility economic analysis of ThinkCancer!: a primary care intervention to expedite cancer diagnosis in Wales
Source: Pilot Feasibility Stud. 2021 Apr 21;7:100. doi: 10.1186/s40814-021-00834-y (PMC8059131; doi:10.1186/s40814-021-00834-y)

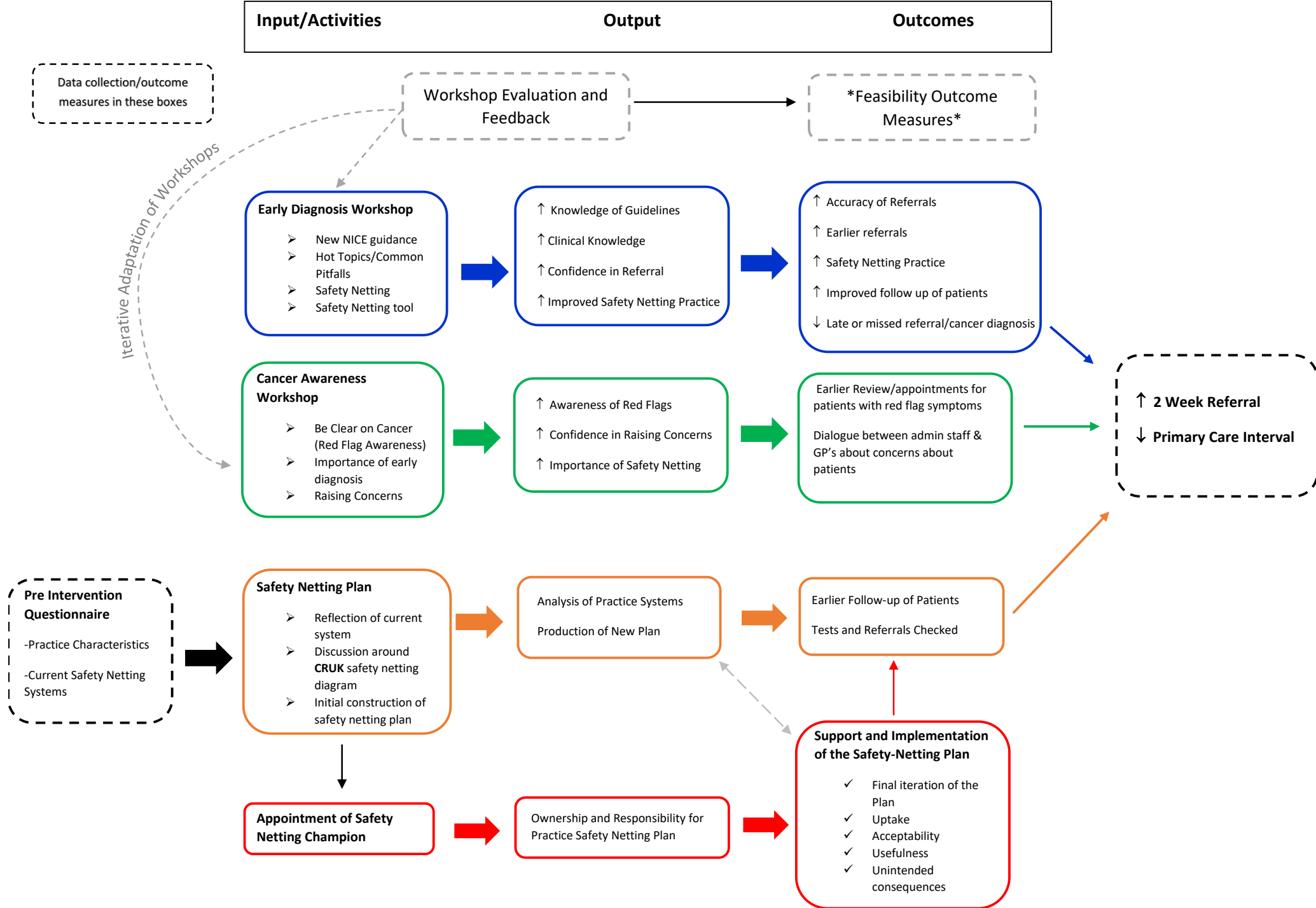

Supplement: Supplementary file 2 — Additional file 2:. ThinkCancer! Logic Model. [file 40814_2021_834_MOESM2_ESM.pdf]

Figure 2: Participant flow diagram

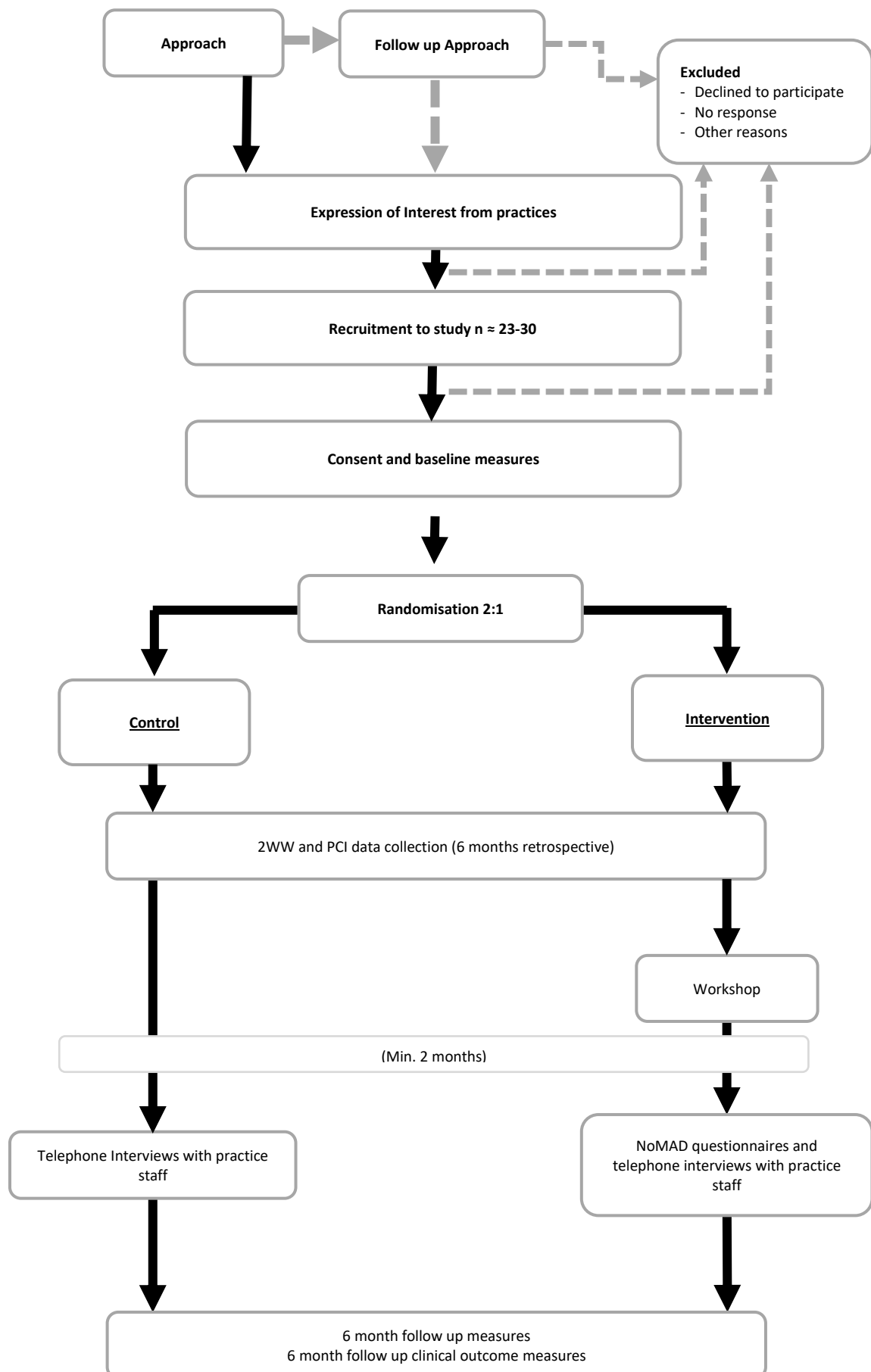

Supplement: Supplementary file 3 — Additional file 3. participant flow diagram. [file 40814_2021_834_MOESM3_ESM.pdf]
