# Supplementary material for: Protocol for a feasibility study incorporating a randomised pilot trial with an embedded process evaluation and feasibility economic analysis of ThinkCancer!: a primary care intervention to expedite cancer diagnosis in Wales
Source: Pilot Feasibility Stud. 2021 Apr 21;7:100. doi: 10.1186/s40814-021-00834-y (PMC8059131; doi:10.1186/s40814-021-00834-y)
Supplement: Supplementary file 4 — Additional file 4:. SPIRIT Checklist. [file 40814_2021_834_MOESM4_ESM.pdf]

SPIRIT Checklist

SPIRIT/CONSORT 2013 Checklist: SPIRIT Checklist merged with CONSORT Extension for pilot/feasibility trials checklist

SPIRIT items that have been replaced by a CONSORT item will be denoted with an *

Section/item	Item No	Description	Addressed on page number
**Administrative information**			
Title	1	Descriptive title identifying the study design, population, interventions, and, if applicable, trial acronym	Title Page (p1)
Trial registration	2a	Trial identifier and registry name. If not yet registered, name of intended registry	p3
	2b	All items from the World Health Organization Trial Registration Data Set	Additional file 1
Protocol version	3	Date and version identifier	Title Page (p1)
Funding	4	Sources and types of financial, material, and other support	p29
Roles and responsibilities	5a	Names, affiliations, and roles of protocol contributors	p29
	5b	Name and contact information for the trial sponsor	p29
	5c	Role of study sponsor and funders, if any, in study design; collection, management, analysis, and interpretation of data; writing of the report; and the decision to submit the report for publication, including whether they will have ultimate authority over any of these activities	p29 (sponsor)<br>p29 (funder)

	5d	Composition, roles, and responsibilities of the coordinating centre, steering committee, endpoint adjudication committee, data management team, and other individuals or groups overseeing the trial, if applicable (see Item 21a for data monitoring committee)	p23
**Introduction**			
Background and rationale	6a	Description of research question and justification for undertaking the trial, including summary of relevant studies (published and unpublished) examining benefits and harms for each intervention	p1
	6b	Explanation for choice of comparators	p4
CONSORT 2a	7a*	Scientific background and explanation of rationale for future definitive trial, and reasons for randomised pilot trial	p5
CONSORT 2b	7b*	Specific objectives or research questions for pilot trial	p6
Trial design	8	Description of trial design including type of trial, allocation ratio, and framework	p7<br>Allocation ratio described on p13
**Methods: Participants, interventions, and outcomes**			
Study setting	9	Description of study settings (eg, community clinic, academic hospital) and list of countries where data will be collected. Reference to where list of study sites can be obtained	p9
Eligibility criteria	10	Inclusion and exclusion criteria for participants. If applicable, eligibility criteria for study centres and individuals who will perform the interventions (eg, surgeons, psychotherapists)	p15
Interventions	11a	Interventions for each group with sufficient detail to allow replication, including how and when they will be administered	p9

	11b	Criteria for discontinuing or modifying allocated interventions for a given trial participant (eg, drug dose change in response to harms, participant request, or improving/worsening disease)	p17
	11c	Strategies to improve adherence to intervention protocols, and any procedures for monitoring adherence (eg, drug tablet return, laboratory tests)	p12
	11d	Relevant concomitant care and interventions that are permitted or prohibited during the trial	p15
Outcomes<br>(CONSORT 6a)	12*	Completely defined pre-specified assessments or measurements to address each pilot trial objective specified in 7, including how and when they were assessed	p11
Participant timeline	13	Time schedule of enrolment, interventions (including any run-ins and washouts), assessments, and visits for participants. A schematic diagram is highly recommended (see Figure)	Figure 2 – p15<br>Additional file 3 (participant flow diagram)
Sample size<br>(CONSORT 7a)	14*	Rationale for numbers in the pilot trial	p14
Recruitment	15	Strategies for achieving adequate participant enrolment to reach target sample size	p14
**Methods: Assignment of interventions (for controlled trials)**			
Allocation:			
Sequence generation	16a	Method of generating the allocation sequence (eg, computer-generated random numbers), and list of any factors for stratification. To reduce predictability of a random sequence, details of any planned restriction (eg, blocking) should be provided in a separate document that is unavailable to those who enrol participants or assign interventions	p17

Allocation concealment mechanism	16b	Mechanism of implementing the allocation sequence (eg, central telephone; sequentially numbered, opaque, sealed envelopes), describing any steps to conceal the sequence until interventions are assigned	p17
Implementation	16c	Who will generate the allocation sequence, who will enrol participants, and who will assign participants to interventions	p17
Blinding (masking)	17a	Who will be blinded after assignment to interventions (eg, trial participants, care providers, outcome assessors, data analysts), and how	p17
	17b	If blinded, circumstances under which unblinding is permissible, and procedure for revealing a participant's allocated intervention during the trial	p17
**Methods: Data collection, management, and analysis**			
Data collection methods	18a	Plans for assessment and collection of outcome, baseline, and other trial data, including any related processes to promote data quality (eg, duplicate measurements, training of assessors) and a description of study instruments (eg, questionnaires, laboratory tests) along with their reliability and validity, if known. Reference to where data collection forms can be found, if not in the protocol	p17
	18b	Plans to promote participant retention and complete follow-up, including list of any outcome data to be collected for participants who discontinue or deviate from intervention protocols	p17
Data management	19	Plans for data entry, coding, security, and storage, including any related processes to promote data quality (eg, double data entry; range checks for data values). Reference to where details of data management procedures can be found, if not in the protocol	p24
Statistical methods	20a	Methods used to address each pilot trial objective whether qualitative or quantitative	p21
	20b	Methods for any additional analyses (eg, subgroup and adjusted analyses)	p22

	20c	Definition of analysis population relating to protocol non-adherence (eg, as randomised analysis), and any statistical methods to handle missing data (eg, multiple imputation)	p22
**Methods: Monitoring**			
Data monitoring	21a	Composition of data monitoring committee (DMC); summary of its role and reporting structure; statement of whether it is independent from the sponsor and competing interests; and reference to where further details about its charter can be found, if not in the protocol. Alternatively, an explanation of why a DMC is not needed	p24
	21b	Description of any interim analyses and stopping guidelines, including who will have access to these interim results and make the final decision to terminate the trial	p13
Harms	22	Plans for collecting, assessing, reporting, and managing solicited and spontaneously reported adverse events and other unintended effects of trial interventions or trial conduct	p21
Auditing	23	Frequency and procedures for auditing trial conduct, if any, and whether the process will be independent from investigators and the sponsor	p23
**Ethics and dissemination**			
Research ethics approval	24	Plans for seeking research ethics committee/institutional review board (REC/IRB) approval	p28
Protocol amendments	25	Plans for communicating important protocol modifications (eg, changes to eligibility criteria, outcomes, analyses) to relevant parties (eg, investigators, REC/IRBs, trial participants, trial registries, journals, regulators)	p23
Consent or assent	26a	Who will obtain informed consent or assent from potential trial participants or authorised surrogates, and how (see Item 32)	p16

	26b	Additional consent provisions for collection and use of participant data and biological specimens in ancillary studies, if applicable	No ancillary studies are planned
Confidentiality	27	How personal information about potential and enrolled participants will be collected, shared, and maintained in order to protect confidentiality before, during, and after the trial	p24
Declaration of interests	28	Financial and other competing interests for principal investigators for the overall trial and each study site	p29
Access to data	29	Statement of who will have access to the final trial dataset, and disclosure of contractual agreements that limit such access for investigators	p29
Ancillary and post-trial care	30	Provisions, if any, for ancillary and post-trial care, and for compensation to those who suffer harm from trial participation	p30
Dissemination policy	31a	Plans for investigators and sponsor to communicate trial results to participants, healthcare professionals, the public, and other relevant groups (eg, via publication, reporting in results databases, or other data sharing arrangements), including any publication restrictions	p25
	31b	Authorship eligibility guidelines and any intended use of professional writers	p25
	31c	Plans, if any, for granting public access to the full protocol, participant-level dataset, and statistical code	p29
**Appendices**			
Informed consent materials	32	Model consent form and other related documentation given to participants and authorised surrogates	Additional files 5 & 6
Biological specimens	33	Plans for collection, laboratory evaluation, and storage of biological specimens for genetic or molecular analysis in the current trial and for future use in ancillary studies, if applicable	Biological specimens will not be collected in this study

*It is strongly recommended that this checklist be read in conjunction with the SPIRIT 2013 Explanation & Elaboration for important clarification on the items. Amendments to the protocol should be tracked and dated. The SPIRIT checklist is copyrighted by the SPIRIT Group under the Creative Commons "Attribution-NonCommercial-NoDerivs 3.0 Unported" license
